# The role of the jasmonate signalling transcription factors MYC2/3/4 in circadian clock-mediated regulation of immunity in Arabidopsis

**DOI:** 10.1098/rstb.2023.0338

**Published:** 2025-01-23

**Authors:** Rageema Joseph, Jessica L. Odendaal, Robert A. Ingle, Laura C. Roden

**Affiliations:** ^1^ Department of Molecular and Cell Biology, University of Cape Town, Rondebosch 7700, South Africa; ^2^ Department of Pathology, Division of Medical Virology, Institute of Infectious Disease and Molecular Medicine, Faculty of Health Sciences, University of Cape Town, Observatory 7925, South Africa; ^3^ Centre for Health and Life Sciences, Coventry University, Coventry CV1 2DS, UK

**Keywords:** *Arabidopsis*, *Botrytis cinerea*, circadian, jasmonic acid, immune response, MYC

## Abstract

Plants are exposed to pathogens at specific, yet predictable times of the day–night cycle. In Arabidopsis, the circadian clock influences temporal differences in susceptibility to the necrotrophic pathogen *Botrytis cinerea*. The jasmonic acid (JA) pathway regulates immune responses against *B. cinerea*. The paralogous basic helix–loop–helix transcription factors MYC2, MYC3 and MYC4 are primary regulators of the JA pathway, but their role in regulating temporal variation in immunity is untested. This study aimed to investigate the roles of the MYC transcription factors in the temporal defence response to *B. cinerea*. We inoculated leaves from wild-type, *myc* single-, double- and triple-knockout mutants, and lines overexpressing *MYC2*, *MYC3* or *MYC4*, with *B. cinerea* at two times of day in constant light, and compared lesion sizes. The presence of MYC2, MYC3 or MYC4 alone was sufficient to maintain temporal variation in susceptibility, but this was abolished in the *myc234* triple-knockout mutant. Constitutive expression of *MYC2*, *MYC3* or *MYC4* abolished time-of-day differences in susceptibility. The data suggest that MYC2, MYC3 and MYC4 function redundantly in regulating temporal defence responses against *B. cinerea* and are a point of convergence between the JA pathway and the circadian clock in Arabidopsis.

This article is part of the Theo Murphy meeting issue ‘Circadian rhythms in infection and immunity’.

## 1. Introduction

Jasmonic acid (JA) and its derivatives (jasmonates) are fatty acid-derived oxylipins that play a wide range of roles in plants, including the co-ordination of developmental pathways, seed germination, fertility and responses to biotic and abiotic stresses including herbivory, pathogen attack and wounding. They function in the fine-tuning of the defence–growth trade-off by acting as integrators of developmental and stress signalling to allocate resources appropriately [1–4]. JA levels show rhythmic variation under a 12 h light/dark (L/D) cycle, peaking in the middle of the day [5]. JA undergoes different enzymatic transformations to produce derivatives with distinct biological functions. For example, the JASMONATE RESISTANT 1 enzyme facilitates the conjugation of JA with isoleucine to form jasmonoyl-L-isoleucine (JA-Ile; [6]), which is considered to be the primary bioactive derivative of jasmonates and is fundamental to JA signalling in Arabidopsis [7].

In the absence of the JA-Ile hormone signal, JA-responsive gene expression is suppressed through the action of one of the 12 members of the JASMONATE ZIM DOMAIN (JAZ) protein family. JAZ proteins inhibit the transcriptional activity of the basic-helix–loop–helix (bHLH) transcription factors (TFs) MYC2, MYC3 and MYC4 that regulate a suite of JA responses [1,2,4,8–10]. JAZ proteins act as negative regulators of these TFs by recruiting the general repressor TOPLESS, leading to histone deacetylation, by competing with Mediator25 for TF binding, or by inhibiting TF binding to DNA [4,11–16]. Bioactive JA-Ile is perceived by the F-box protein CORONATINE INSENSITIVE 1 (COI1; [17]), which associates with Skp, Cullin and Rbx, forming an SCF^COI1^ complex with E3 ubiquitin ligase activity [18–21]. Upon JA-Ile binding, the SCF^COI1^ complex recruits JAZ repressors for ubiquitination and degradation by the 26S proteasome, alleviating the repression imposed on the MYC TFs, which then activate the expression of primary JA response genes [7,11,12,22,23]. Although there is only a single receptor for JA-Ile, viz*.* COI1, it is thought that the functional specificity of signalling may be due to distinct JAZ–transcription factor interactions and the spatiotemporal abundance of specific regulatory components [3].

Although MYC2 has been classified as a negative regulator of defence against the necrotrophic fungi *Botrytis cinerea, Plectosphaerella cucumerina* and *Fusarium oxysporum* [8,24], little is known about the contributions of the closely related proteins MYC3 and MYC4. The MYC TFs displayed a high degree of similarity with respect to their modular structure, stability and interactions with *cis*-regulatory elements and JAZ proteins [2,8,10,25–27]. This suggests that the MYC TFs may regulate common processes. Despite the high degree of homology, there are differences in the regulatory contributions of individual MYC proteins, and these differences are dependent on the JA-responsive process being investigated. For example, while defence against generalist herbivores is achieved through the combined action of MYC2, MYC3 and MYC4, MYC3 and MYC4 play a more prominent role [2]. The differences in the regulatory roles may be due to differences in spatial expression patterns, with *MYC3* and *MYC4* preferentially expressed in aerial tissues and *MYC2* in the roots [2,10], and/or complex formation as MYC2, MYC3 and MYC4 can form both homo- and heterodimers [2].

Plants encounter diverse pathogens at specific, yet predictable times of the day–night cycle. The circadian clock, an endogenous timing mechanism, allows plants to anticipate recurring changes in pathogen abundance, and coordinate their physiology and behaviour to complement environmental changes [28]. *Botrytis cinerea* conidia exhibit diurnal rhythms of spore production and release, with an increase in temperature after sunrise prompting the release of conidia. Consequently, spores are more abundant in natural environment from the morning to afternoon and pose a greater threat to plant fitness [29–31]. It is well known that JA signalling is a key host response against necrotrophic pathogen infection [32], and it has previously been implicated in the temporal regulation of immunity to *B. cinerea*. Functional enrichment analysis of the 219 genes that showed enhanced induction in Arabidopsis 18 h post inoculation with *B. cinerea* spores at subjective dawn versus night revealed enrichment for JA-responsive genes, and temporal variation in immunity to this pathogen was lost in a *jaz6* mutant [33]. There are several potential points of intersection between the circadian clock and the JA pathway. Mechanisms of circadian regulation include the phasing of JA biosynthesis and accumulation [5], rhythmic expression of JA-responsive genes [34] and differential regulation of the magnitude of defence responses at different times of the day [33]. Furthermore, the clock component TIME FOR COFFEE (TIC) interacts with MYC2, prompting proteosomal degradation of this TF, and also represses *COI1* transcription in the evening [28,35]. There is also evidence that JA signalling impacts clock activity; the amplitude of expression of core clock genes, including *CCA1*, *LHY* and *TOC1*, is reduced in response to both *B. cinerea* infection [36] and methyl-JA application [37].

As primary regulators of the JA pathway, the MYCs represent a possible point of convergence between the circadian clock and the JA pathway in regulating defence responses. Thus, the primary aims of this work were to investigate the roles of the MYC TFs in defence responses against *B. cinerea* and their possible roles in the crosstalk between the circadian clock and the JA pathway in Arabidopsis.

## 2. Material and methods

### (a) Plant material

All Arabidopsis knockout and over-expressor lines used in this study have previously been characterized. *MYC* over-expressor lines and the *myc3* GABI-Kat_445B11 line [10] were obtained from John Browse (Washington State University). The *myc4* GABI-Kat_491E10 and the double and triple *myc*-knockouts [2] were provided by Roberto Solano (Spanish National Centre for Biotechnology). The previously characterized *myc2* line (SALK_017005; [24]) was obtained from the Nottingham Arabidopsis Stock Centre, and T-DNA insert position and suppression of *MYC2* expression were confirmed prior to use.

### (b) Plant growth conditions

Arabidopsis seeds were sown onto a 1 : 1 mixture of peat (Jiffy Products, Norway) and vermiculite and covered in cling wrap. After stratification in the dark at 4°C for 3 days, they were transferred to 16 h/8 h (L/D) cycles at 22°C, under cool white fluorescent light, 80–100 µmol m^−2^ s^−1^. After four weeks, plants were transferred to constant light (LL) during their subjective day period for at least 24 h prior to infection.

### (c) *Botrytis cinerea* infection assays

*Botrytis cinerea* pepper isolate [38] was propagated in the dark on low-sugar canned apricot halves at 25°C for 10–14 days prior to use of the spores. Inoculations were carried out on detached leaves and lesions measured as previously described [39]. Lesion areas were transformed by square-rooting (to satisfy assumption of homogeneity of variance) and data were analysed in Statistica (v. 13). Two-way analysis of variance (ANOVA) was used to test for a significant effect of time of inoculation, plant genotype and an interaction between these two factors on lesion area in each experiment. The *F*-ratio, *p*-value and effect size (partial *η*^2^) for all factors significant at *p* < 0.05 are provided in the figure legends. If one or more factors were significant at *p* < 0.05, a Fisher least significant difference (LSD) *post hoc* test was then used to identify mean lesion area values significantly different at *p* < 0.05.

### (d) Analysis of *MYC* expression in microarray datasets

Signal intensities were extracted from normalized microarray datasets obtained from the NCBI Gene Expression Omnibus (GEO) repository: GSE3416 Diurnal regulation—RNA extracted at 4 h intervals starting at the end of the night from plants entrained to 12 h L/D cycles at constant temperature of 20°C [40]; and GSE5612 Circadian regulation—plants entrained to 12 h L/D cycle were exposed to continuous light and RNA sampled at 4 h intervals over 48 h beginning 2 h after subjective dawn on second day of LL (made public in 2007 by K. Edwards, A. Millar, H. Townsend, Z. Emmerson, B. Schildknecht). Signal intensities for each MYC TF within each experiment were standardized (mean value of 0 and s.d. of 1).

## 3. Results

### (a) MYC2, MYC3 and MYC4 play redundant roles in temporal regulation of immunity against *Botrytis cinerea*

**Figure 1.**
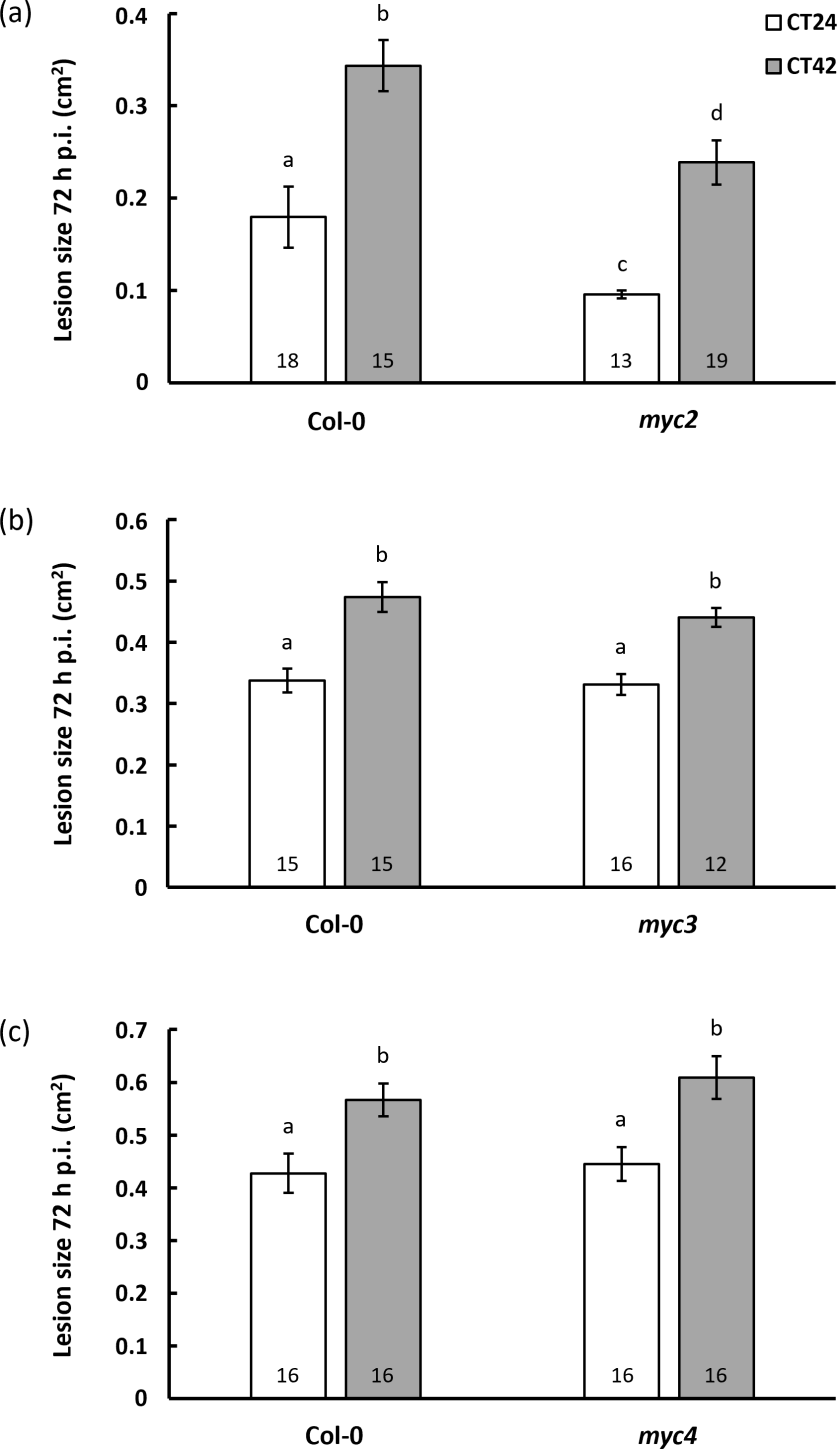
Temporal variation in immunity to *Botrytis cinerea* is unaffected in *myc* single-knockout mutants. Detached leaves from four-week-old plants were inoculated with *B. cinerea* spores at CT24 (subjective dawn) or CT42 (subjective night) under constant light conditions, and lesion size measured at 72 h post infection (p.i.). Data shown are mean values ± s.e.m., with the number of leaves (from independent plants) inoculated indicated within the bars. Time of inoculation (*F* = 47.74, *p* < 0.001, partial *η*^2^ = 0.44) and plant genotype (*F* = 14.91, *p* < 0.001, partial *η*^2^ = 0.20) had a significant effect on lesion size in a two-way ANOVA in the Col-0 versus *myc2* comparison (*a*) while only time of inoculation was significant in the Col-0 versus *myc3* (*b*) (*F* = 29.69, *p* < 0.001, partial *η*^2^ = 0.35) and *myc4* (*c*) (*F* = 18.13, *p* < 0.001, partial *η*^2^ = 0.23) analyses. Mean lesion sizes with different letters are significantly different (*p* < 0.05) as determined by Fisher LSD *post hoc* test. Results shown are from one experiment representative of three independent experiments.

To determine whether *MYC2*, *MYC3* and *MYC4* are required for temporal variation in susceptibility to *B. cinerea*, we first challenged leaves from single-knockout lines with *B. cinerea* spores at times CT24 and CT42. As was the case for Col-0, lesion areas on the *myc2*, *myc3* and *myc4* mutants were significantly larger on leaves inoculated at CT42 versus CT24 (figure 1), i.e. the temporal variation in susceptibility persisted in all three mutants. However, some differences in lesion sizes were apparent between the mutant lines, with *myc2* displaying significantly smaller lesions than Col-0 following inoculation at both CT24 and CT42, in line with the previously reported reduced susceptibility to the necrotrophic pathogen *F. oxysporum* [25] and *B. cinerea* [8,41,42]. In contrast, lesion sizes in the *myc3* and *myc4* mutants were not significantly different from those in Col-0 at either time of inoculation (figure 1).

**Figure 2.**
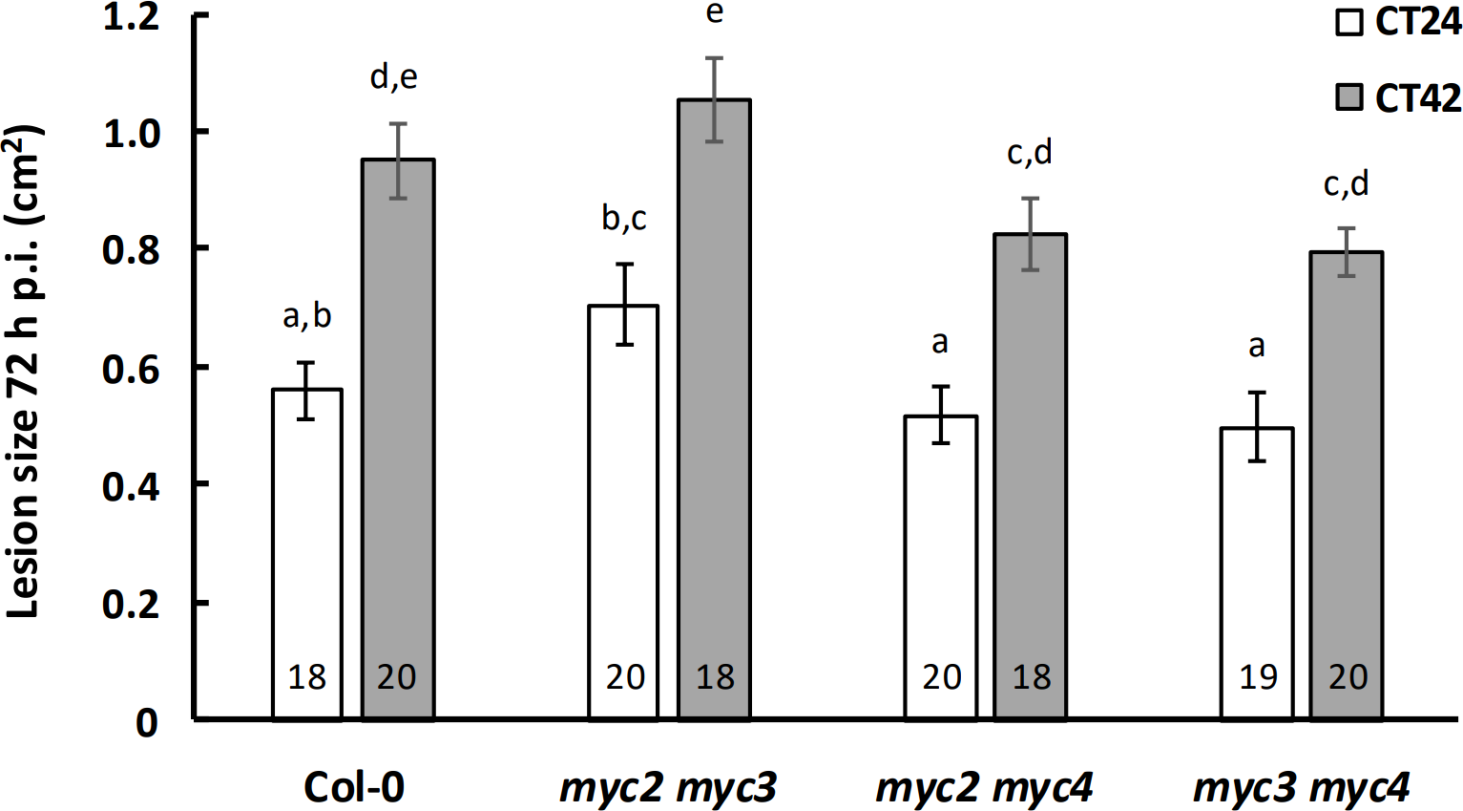
Temporal variation in immunity to *Botrytis cinerea* is unaffected in *myc* double-knockout mutants. Detached leaves from four-week-old plants were inoculated with *B. cinerea* spores at CT24 (subjective dawn) or CT42 (subjective night) under constant light conditions, and lesion size was measured at 72 h p.i. Data shown are mean values ± s.e.m., with the number of leaves (from independent plants) inoculated indicated within the bars. Time of inoculation (*F* = 70.59, *p* < 0.001, partial *η*^2^ = 0.33) and plant genotype (*F* = 5.82, *p* < 0.001, partial *η*^2^ = 0.11) had a significant effect on lesion size in a two-way ANOVA. Mean lesion sizes with different letters are significantly different (*p* < 0.05) as determined by Fisher LSD *post hoc* test. Results shown are from one experiment representative of three independent experiments.

**Figure 3.**
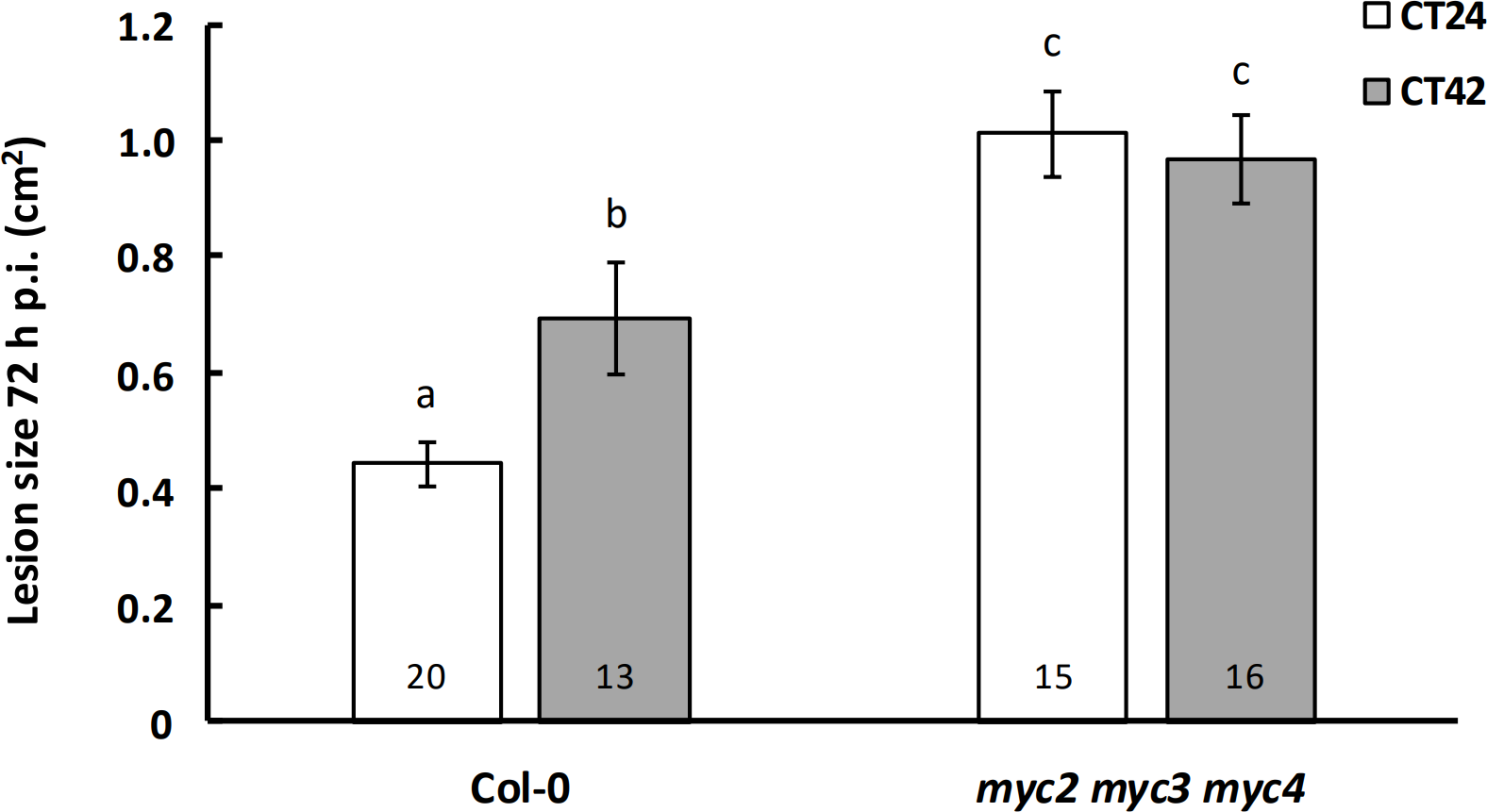
Temporal variation in immunity to *Botrytis cinerea* is abolished in a *myc2 myc3 myc4* triple-knockout mutant. Detached leaves from four-week-old plants were inoculated with *B. cinerea* spores at CT24 (subjective dawn) or CT42 (subjective night) under constant light conditions, and lesion size was measured at 72 h p.i. Data shown are mean values ± s.e.m., with the number of leaves (from independent plants) inoculated indicated within the bars. Plant genotype (*F* = 41.06, *p* < 0.001, partial *η*^2^ = 0.41) and plant genotype × time of inoculation (*F* = 5.31, *p* = 0.025, partial *η*^2^ = 0.08) had a significant effect on lesion size in a two-way ANOVA. Mean lesion sizes with different letters are significantly different (*p* < 0.05) as determined by Fisher LSD *post hoc* test. Results shown are from one experiment representative of three independent experiments.

We next tested the *myc2 myc3*, *myc2 myc4* and *myc3 myc4* double mutants for temporal variation in susceptibility to *B. cinerea*. All three lines displayed the expected increased susceptibility to this pathogen when inoculated at subjective night (figure 2). In contrast to the *myc2* single mutants, no significant difference in lesion area was detected between Col-0 and either the *myc2 myc3* or *myc2 myc4* double mutants at either time of inoculation (figure 2). Finally, we analysed lesion sizes in a *myc2 myc3 myc4* (*myc234*) triple mutant inoculated at CT24 and CT42. Variation in susceptibility to *B. cinerea* was abolished at these two time points in this line, and lesion sizes were significantly greater than those in Col-0 72 h p.i. at both CT24 and CT42 (figure 3). These data suggest that the presence of any one of MYC2, MYC3 or MYC4 is sufficient to allow temporal regulation of immunity against this pathogen in Arabidopsis. However, the different susceptibility phenotypes observed in the *myc2*, *myc2 myc3*, *myc2 myc4* and *myc234* lines suggests that these TFs might play different roles in immunity.

### (b) Analysis of MYC over-expressor lines

**Figure 4.**
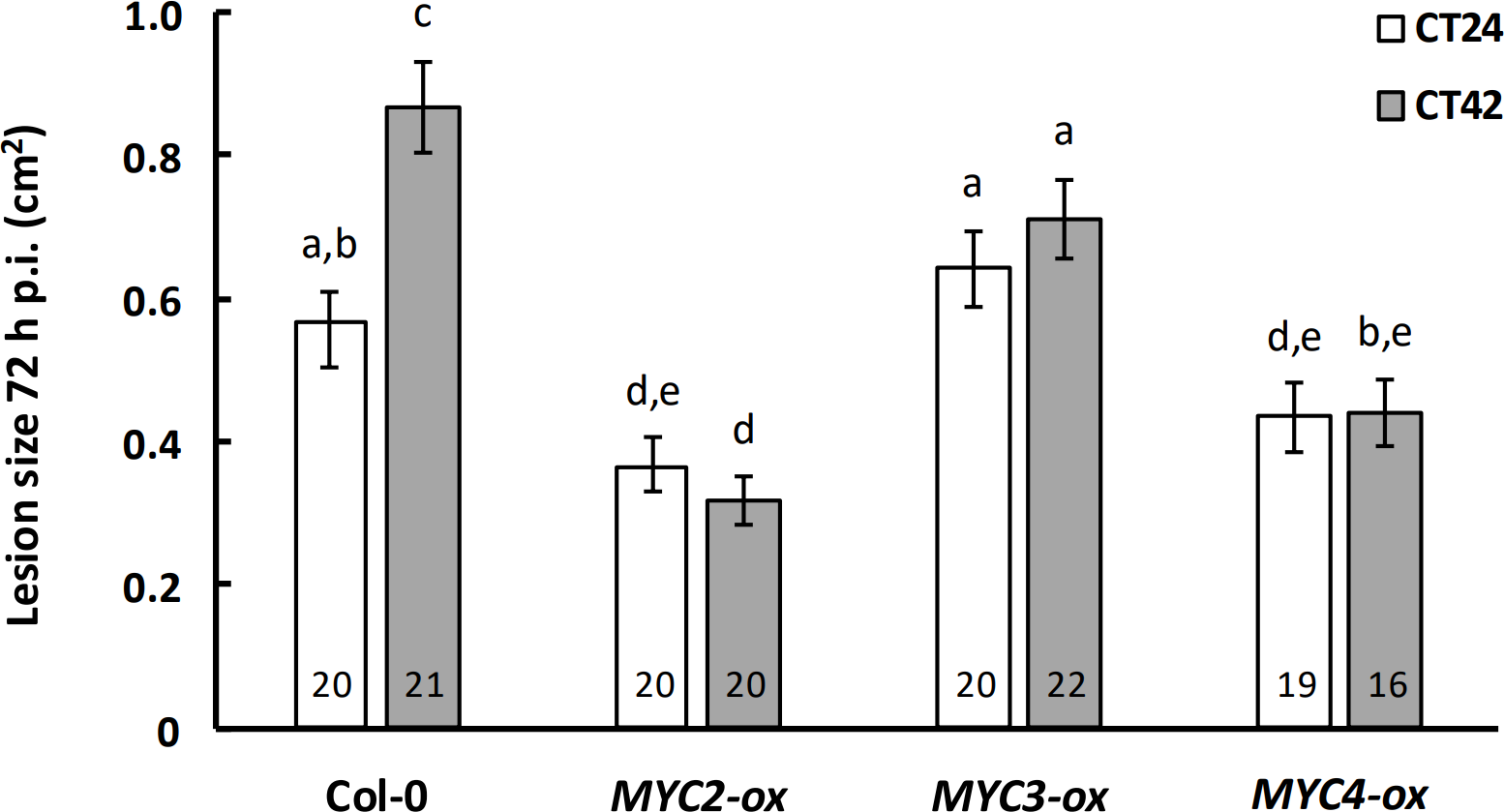
Over-expression of a single *MYC* transcription factor is sufficient to abolish temporal variation in immunity to *Botrytis cinerea*. Detached leaves from four-week-old plants were inoculated with *B. cinerea* spores at CT24 (subjective dawn) or CT42 (subjective night) under constant light conditions, and lesion size measured at 72 h p.i. Data shown are mean values ± s.e.m., with the number of leaves (from independent plants) inoculated indicated within the bars. Plant genotype (*F* = 29.93, *p* < 0.001, partial *η*^2^ = 0.37), time of inoculation (*F* = 3.97, *p* = 0.048, partial *η*^2^ = 0.03) and plant genotype × time of inoculation (*F* = 3.79, *p* = 0.012, partial *η*^2^ = 0.07) had a significant effect on lesion size in a two-way ANOVA. Mean lesion sizes with different letters are significantly different (*p* < 0.05) as determined by Fisher LSD *post hoc* test. Results shown are from one experiment representative of three independent experiments.

To understand better the roles played by the MYC TFs in regulation of immunity against *B. cinerea*, we analysed the disease-resistance phenotypes in lines overexpressing *MYC2*, *MYC3* or *MYC4*. We observed that temporal variation in susceptibility to this pathogen was abolished irrespective of which specific MYC TF was overexpressed (figure 4). However, the degree of susceptibility to *B. cinerea* infection varied between the lines. Increased resistance to *B. cinerea* was observed in both the *MYC2-ox* and *MYC4-ox* lines, with lesion sizes 72 h p.i. at CT24 and CT42 significantly smaller than those observed in Col-0 at CT24. In contrast, overexpression of *MYC3* resulted in lesion sizes at C24 and CT42 that were not significantly different from those observed in Col-0 after inoculation at CT24 (figure 4).

### (c) Analysis of *MYC* expression under diurnal and constant conditions

**Figure 5.**
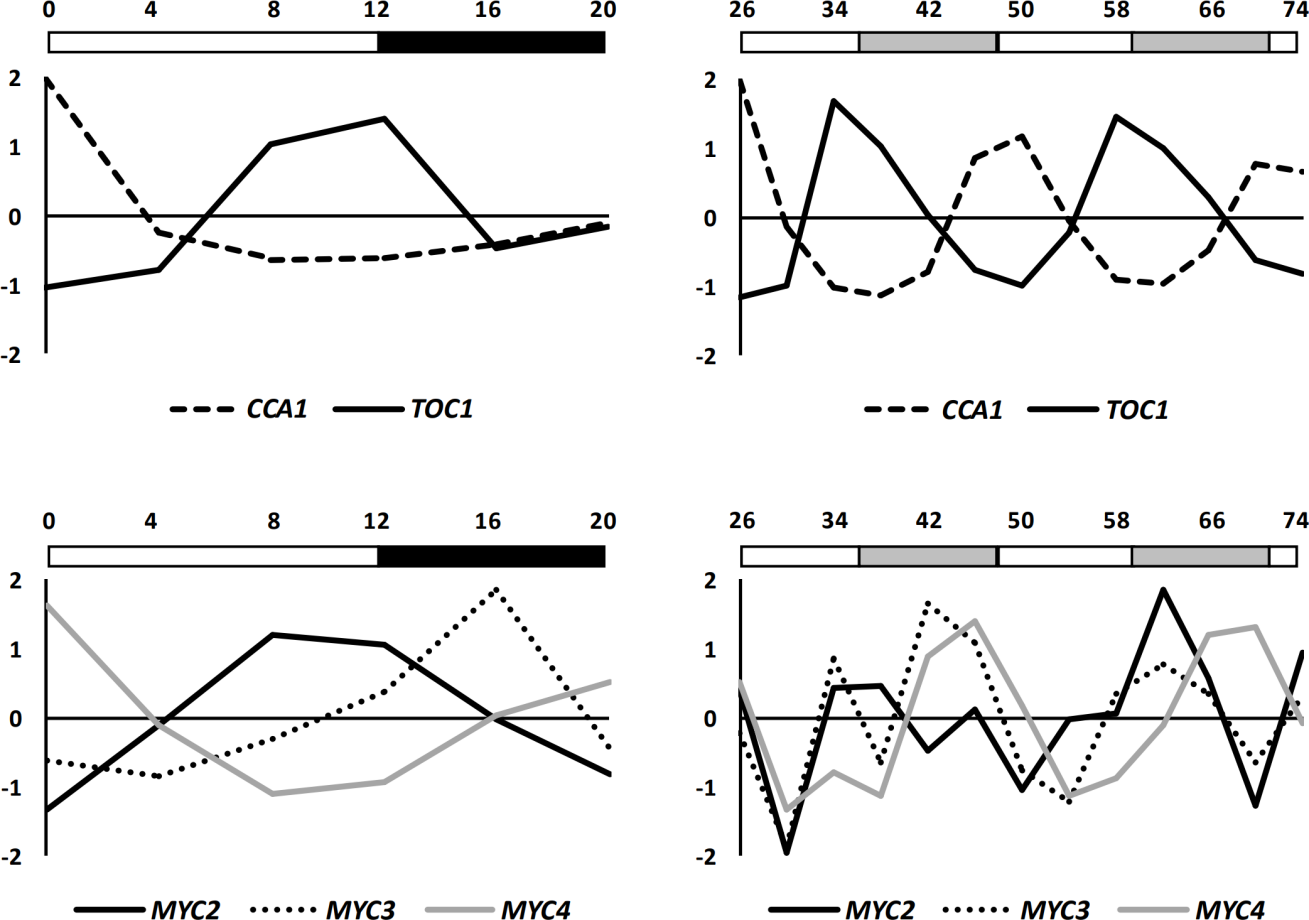
Circadian regulation of *MYC2*, *MYC3* and *MYC4* expression. Profiles for transcripts of central oscillator genes *CCA1* and *TOC1* are shown in top panels for comparison. Expression profiles for all transcripts were generated from standardised signal intensities downloaded from microarray datasets in the NCBI GEO database (Diurnal, GSE3416 and Circadian, GSE5612). Day and night are indicated by white and black boxes, respectively, above each plot (diurnal) and subjective day and night are indicated by white and grey boxes, respectively, above each plot (circadian). Time in h indicated on x-axes.

Loss of temporal variation in susceptibility to *B. cinerea* in the *myc234* and *MYC-ox* plants indicate that these TFs might be a point of interaction between the circadian clock and defence network in plants. One possible way in which this might occur is via clock regulation of transcriptional activity of the genes encoding these TFs. As a first step to testing this, we interrogated publicly available microarray datasets where RNA had been sampled every 4 h in plants grown under a 12 h L/D cycle for 20 h (Diurnal, GSE3416) or every 4 h for 48 h in plants entrained to a 12 h L/D cycle and transferred to LL conditions 24 h prior to the start of the experiment (Circadian, GSE5612). Under L/D conditions, *MYC2*, *MYC3* and *MYC4* expression peaked late in the light period, at dusk, and at the end of the night, respectively (figure 5). Under LL conditions, the microarray data indicated that *MYC2*, *MYC3* and *MYC4* exhibited rhythmic expression, with a slight shift in the acrophase of *MYC2* relative to *MYC3* and *MYC4* (figure 5).

## 4. Discussion

When leaves were inoculated with *B. cinerea* spores at different times of the day, the temporal variation in response to infection observed in wild-type plants was retained in the single (figure 1) and double (figure 2) *myc*-knockout lines, with smaller lesion sizes observed following inoculation at subjective dawn compared with subjective night. Although JAZ2 modulates stomatal dynamics during bacterial invasion [43], and *B. cinerea* is reported to exploit apertures such as stomata and wounds for host entry [44], hyphal penetration of the plant cuticle is recognized as the dominant means of entry [31]. Furthermore, we have previously observed that *B. cinerea* hyphae showed no propensity to utilize open stomata on Arabidopsis leaves as a means of entry [33]. The observed differences in susceptibility were therefore unlikely a consequence of clock-regulated or MYC-mediated stomatal regulation. Similarly, while a role for the *B. cinerea* circadian clock in temporal variation of pathogen virulence has previously been reported [45], this is highly unlikely to explain the temporal variation in lesion size in these experiments as we cultured the fungus in constant darkness, and at constant temperature, thus providing no entrainment signal for the fungal clock prior to inoculation.

The variation in susceptibility to *B. cinerea* infection at CT24 versus CT42 was completely abolished in the *myc234* triple knockout and was accompanied by increased susceptibility to the pathogen (figure 3). These data suggest that the MYCs are required for defence against *B. cinerea* and that depletion of all three MYCs renders plants unable to maintain temporal regulation of defence responses to the necrotrophic pathogen. As we did not carry out a time series of infections across a full 24 h period, an alternative possibility for the lack of difference in susceptibility in the *myc234* triple-knockout line could be due to a phase shift that resulted in the inoculation times occurring in the midpoint of the oscillation. Different intrinsic circadian periods could result in a phase difference between the wild-type and the *myc234* triple-knockout line in LL. However, analysis of circadian leaf movement rhythms (electronic supplementary material, figure S1) indicated that there was no significant difference between the periods of Col-0 and the *myc234* triple-knockout line (24.3 h versus 24.0 h; Student’s *t*‐test *p* = 0.44), suggesting that this was not a factor here. Future work to elucidate the crosstalk between the circadian clock and the JA pathway in Arabidopsis might include analysis of circadian clock gene expression in the *MYC*-knockout and over-expression lines, and investigation of *MYC* gene expression in plants that lack functional circadian clocks.

The MYCs are clearly redundant, however, with respect to temporal regulation of plant responses to *B. cinerea*, as the presence of a single MYC and/or a combination of two MYCs is sufficient to sustain the time-of-day variation in susceptibility to *B. cinerea*. While *MYC2* expression is highest in roots, it is not restricted to this tissue type and is expressed at similar levels to that of *MYC3* and *MYC4* in adult leaves (the developmental stage used here; [2], ePlant, accessible at bar.utoronto.ca/eplant/). It is clear that *MYC2* does contribute towards resistance against *B. cinerea* in aerial plant tissues since the *myc3 myc4* double mutant did not display enhanced susceptibility to this pathogen relative to Col-0, while the *myc234* triple mutant did. This observation is at odds with the prevailing view of MYC2 as a negative regulator of immunity to necrotrophic pathogens in Arabidopsis, as is the enhanced resistance phenotype of the *MYC2*-ox line. It has been suggested that the enhanced resistance of *myc2* to necrotrophic pathogens might result from the relief of repression by MYC2 on the transcriptional activity of the key ethylene signalling TF EIN3 [46]. However, MYC3 and MYC4 also interact with EIN3 [42], so it is unclear why *myc3* and *myc4* single mutants would not also show enhanced resistance. Furthermore, the loss of an additional MYC TF in the *myc2* background might be expected to enhance this effect, yet this is not the case and neither the *myc2 myc3* nor *myc3 myc4* double mutant show any difference in susceptibility to *B. cinerea* compared with wild-type plants. It seems highly likely that the regulation of JA-mediated defence responses against necrotrophic pathogens by the MYC TFs is more nuanced than is generally believed.

Over-expression of a single MYC TF was sufficient to abolish the time-of-day variation in susceptibility to *B. cinerea* infection , suggesting that this renders the plants insensitive to clock-derived signals. Despite the high degree of structural and functional homology, constitutive expression of *MYC2* and *MYC4* had more dramatic effects on susceptibility to *B. cinerea* than did that of *MYC3* (figure 4). While lesion sizes following inoculation at CT42 were significantly smaller than those in Col-0 in all three *MYC*-ox genotypes, only *MYC2*-ox and *MYC4*-ox plants had smaller lesions following infection at CT24. Although it is possible that this reflects a degree of functional divergence between MYC3 versus MYC2 and MYC4, we cannot exclude the possibility that it might simply reflect different levels of transgene expression between the over-expressor lines.

Diurnal and circadian expression of MYC2 protein levels has been demonstrated, with levels peaking at ZT8 under a 12 L/D dark cycle [28,35]. While similar experiments were not conducted to establish whether MYC3 and MYC4 proteins are subject to the same regulation, it is likely that this occurs. We thus hypothesize that in wild-type plants, rhythmic oscillations in MYC protein levels over a 24 h period lead to time-of-day differences in MYC occupancy at the promoters of defence genes, driving variation in susceptibility to *B. cinerea*. In the absence of MYC2, MYC3 and MYC4, no MYC proteins are present to bind to the promoters of target genes and regulate their expression in a time-of-day-dependent manner. In the absence of one or more of the *MYC* genes, rhythmic expression and accumulation of the remaining paralogue(s) modulate time-of-day differences in susceptibility by binding to the promoters of target genes only at certain times of the day–night cycle. In the over-expressor lines, increased levels of MYC proteins saturate the promoters of target genes throughout the day–night cycle, which abolishes temporal variation in susceptibility (figure 4).

Within the circadian clock, the sequential expression of TIMING OF CAB EXPRESSION 1 (TOC1) or PSEUDO-RESPONSE REGULATOR1 (PRR1), PRR9, PRR7 and PRR5 from early daytime to late at night limits the expression of *CCA1*/*LHY* to the early morning [47–49]. The sequential rhythmic expression of MYC paralogues observed in wild-type plants (figure 5), from late afternoon to early morning, may similarly contribute towards gating of JA-mediated defence, so providng the necessary and appropriately timed surveillance and defence responsiveness for the plant to necrotrophic pathogens such as *B. cinerea*. This suggests that circadian regulation of defences against necrotrophic pathogenesis is mediated by the MYC transcription factors.

## Data Availability

Pathogen assay data are available at [50]. Supplementary material is available online [51].
